# Incidence and Risk Factors of Dry Eye in Children and Adolescents With Diabetes Mellitus: A 3-Year Follow-Up Study

**DOI:** 10.3389/fmed.2021.760006

**Published:** 2021-11-29

**Authors:** Zhangling Chen, Ying Xiao, Yu Qian, Qiurong Lin, Zhaoyu Xiang, Lipu Cui, Jiaqi Sun, Sicong Li, Xinran Qin, Chenhao Yang, Haidong Zou

**Affiliations:** ^1^Department of Ophthalmology, Shanghai General Hospital of Nanjing Medical University, Shanghai, China; ^2^Department of Ophthalmology, Shanghai Songjiang District Central Hospital, Shanghai, China; ^3^Department of Ophthalmology, Children's Hospital of Fudan University, Shanghai, China; ^4^Department of Ophthalmology, Shanghai General Hospital, Shanghai Jiao Tong University School of Medicine, Shanghai, China; ^5^Shanghai Key Laboratory of Fundus Diseases, Shanghai, China; ^6^Shanghai Eye Diseases Prevention & Treatment Center, Shanghai Eye Hospital, Shanghai, China; ^7^National Clinical Research Center for Eye Diseases, Shanghai, China; ^8^Shanghai Engineering Center for Precise Diagnosis and Treatment of Eye Diseases, Shanghai, China

**Keywords:** children and adolescents, diabetes, dry eye, morbidity, risk factors

## Abstract

**Purpose:** To investigate the incidence and risk factors of dry eye in children with diabetes mellitus (DM) over a period of 3 years.

**Methods:** Children and adolescents with DM (age: 3–14 years) from the Shanghai Children and Adolescent Diabetes Eye (SCADE) study cohort who did not have dry eye in January 2018 were followed-up for 3 years and re-examined in January 2021, and the incidence rate and risk factors for dry eye were calculated.

**Results:** Forty children and adolescents with DM came for follow-up in 2021. Nine of them were diagnosed with dry eye, resulting in a 3-year incidence rate of 22.5% and an annual mean incidence rate of 7.5% for dry eye. Univariate regression analysis confirmed that decreased corneal sensation (OR [Odds Ratio] = 25.60; 95%CI [Confidence Interval] = 1.31~501.69; *P* = 0.03) was the risk factor for dry eye incidence. Long course of DM (OR = 1.80; 95%CI = 0.96~3.38; *P* = 0.07), eye pain (OR = 12.27; 95%CI = 0.65~231.48; *P* = 0.09), and dry eye in parents (OR = 15.99; 95%CI = 0.76~337.75; *P* = 0.08) may interfere with the incidence of dry eye in them.

**Conclusions:** The incidence of dry eye in children and adolescents with DM is high.

## Introduction

The prevalence of diabetes mellitus (DM) is increasing every year globally. The International Diabetes Federation revealed that in 2011, the number of DM patients worldwide was 370 million, and the number may reach ~550 million by 2030, of which 80% will be from developing countries (1). In 2017, the numbers of children and adolescents (age: <20 years) with type 1 DM in the world and China were 1,106,500 and 47,000, respectively (2). The risk of chronic eye disease always increases with the progression of DM, for example, dry eye, which is a multifactorial disease of the tear and eyeball surface, causing ocular discomfort, visual impairment, tear osmotic pressure increase, and ocular surface inflammation (3). Risk factors for dry eye in the adults include reduced lacrimal gland secretion, changes in tear composition, instable tear film, decreased density of the corneal and conjunctival nerves, reduced number of conjunctival goblet cells, and meibomian gland dysfunction (4–6). The worldwide prevalence of dry eye is ~7–33% (7). Manaviat et al. (8) found that dry eye symptoms happened in 54.3% of the adult patients with type 2 DM, much higher than that of the normal individuals.

Previous epidemiology studies of dry eye in children with DM only reported the prevalence (9, 10), and found the relationship between dry eye and fasting blood glucose, HbA1c values (11), and ocular surface impairment; (9, 12, 13) however, no study has investigated the incidence of dry eye in children with DM or without. In 2018, our team established the Shanghai Children and Adolescent Diabetes Eye Study (SCADE Study) cohort, then we found that the prevalence of dry eye was much higher in children with DM (28.95%) than in normal children (5.00%) in 2018 (10). Since then, we followed these children annually. In the present study, the incidence rate and risk factors of dry eye in them over a 3-year period time are reported.

## Methods

### Research Objective

The participants in the SCADE study cohort without dry eye in January 2018 came for follow up in January 2021 were enrolled in the present study. Inclusion criteria were set as follows: (1) providing written informed consent; (2) aged 6–17 years in 2021; (3) diagnosed with type 1 DM; and (4) fulfill the questionnaire and underwent all eye examinations. Exclusion criteria were set as follows: (1) other ocular and systemic diseases that affect tear secretion and quality, such as eyelid diseases (trichiasis, eyelid eversion, ptosis, and eyelid incompleteness) and conjunctival diseases (acute and chronic infectious conjunctivitis, allergic conjunctivitis, and conjunctival calculus); (2) a history of eye trauma within 6 months; (3) a history of eye surgery within 6 months; (4) severe eye complications caused by DM; (6) receiving eye treatment with eye drops; and (7) with systemic diseases such as systemic lupus erythematosus, Sjogren's syndrome, and Grave's disease. This study was conducted in accordance with the ethical standards of the Helsinki Declaration, and all participants (or their guardians) provided informed consent. This study was approved by the Institutional Review Committee and the Ethics Committee of Shanghai General Hospital, Shanghai Jiao Tong University School of Medicine (approval number: 2018KY209).

### Questionnaires and Examination Items

We used two questionnaires: the Ocular Surface Disease Index (OSDI) and Dry Eye Questionnaire (DEQ), including questions on name, sex, date of birth, height, weight, cell phone number, address, date of examination, presence of diabetes, diabetes type, presence of diabetes complications, history of eye disease (trichiasis and allergic conjunctivitis), ocular trauma, ocular surgery, or medication, presence of eye discomfort (foreign body sensation, fatigue, pain force fluctuations, and frequent blinking), use of contact lens, reading and writing posture, presence of sleep disorders, mental status, dietary preferences, use of air conditioner, passive smoking, daily time spent on electronic screens, refractive errors, and dry eye in parents.

The following examinations were conducted: (1) The best-corrected visual acuity was measured using the LogMAR visual acuity chart (Wenzhou Xingkang, Zhejiang, China), which meets international standards. Cyclopentolate hydrochloride eye drops (Alcon, Belgium) were used for dilation. Refraction was measured using a computerized automatic optometry device (KR-8900, Topcon, Japan), and the final refraction was measured using a comprehensive optometry device (CV-5000, Topcon). (2) A slit-lamp biomicroscope (SL130, Zeiss, Germany) was used to examine the eyelid, conjunctiva, cornea, anterior chamber, iris, pupil, lens, tear film rupture time, and corneal fluorescein staining. (3) Qualitative examination of corneal sensation was performed using a long and thin cotton thread that was twisted out from the tip of a disinfected cotton swab by bending the tip on a cotton stick at 45°. The cornea of the eye under examination was approached from the side with the tip of the cotton thread and gently touched. Patients with normal corneal sensation had immediate blink reflex or perception. (4) The Schirmer I test was performed to assess tear secretion without superficial anesthesia. (5) Biometric examination (IOL Master 700, Zeiss, Germany) was performed to determine the ocular axis, radius of corneal curvature, anterior chamber depth, lens thickness, central corneal thickness, and corneal diameter. (6) A non-contact tonometer (NT-510, NIDEK, Japan) was used to measure the intraocular pressure (IOP) in both eyes. (7) Corneal endothelium counter (TOPCON, SP-2000P) was used to record the number of corneal endothelia in both eyes.

### Dry Eye Diagnosis Index

Tear secretion test (Schirmer *I*-test): The tip of the Schirmer paper (Jingming, Tianjin, China) (5 × 35 mm) was folded into the conjunctival sac, the eyes closed normally, and the length of the tear soaked paper was measured after 5 min.

Break-up Time (BUT) measurement: The saline moistened fluorescein paper (Jingming, Tianjin) was lightly touched to the conjunctiva of the patients; consequently, patients blinked three to four times, spreading the fluorescein evenly on the cornea. The time from the last blink of the eye to the appearance of the first black spot on the corneal surface was the tear film rupture time, which was measured three times to obtain the average value.

Cornea fluorescein staining (CFS): The cornea was observed after uniform staining with fluorescein. No corneal staining was recorded as negative, whereas presence of corneal staining was considered positive.

### Diagnostic Criteria

According to the 2020 Chinese expert consensus on examination and diagnosis of dry eye (14), diagnosis of dry eye can be made based on the following criteria: (1) the patient complaining of one of the subjective symptoms such as eye dryness, foreign body sensation, burning sensation, fatigue, discomfort, redness, and fluctuation of vision and having an OSDI of ≥ 13 points and a BUT of ≤ 5 s or a Schirmer *I*-test value of ≤ 5 mm/5 min. (2) Patients with dry eye-related symptoms, an OSDI score of ≥ 13 points, a BUT of > 5 s and ≤ 10 s or a Schirmer I test value of > 5 mm/5 min and ≤ 10 mm/5 min, and positive staining with fluorescein sodium. Any eye meeting one of the aforementioned criteria was diagnosed as dry eye.

### Quality Control

All children were examined at the Shanghai Eye Disease Prevention & Treatment Center. All eye examinations were performed by the same trained ophthalmologist to ensure consistency in the results.

### Statistical Analysis

IBM SPSS Statistics 22.0 statistical software was used for analysis. The measurement data were tested for K-S normality and the measurement data with normal distribution were presented as mean ± standard deviation and an independent sample *t-test* was performed for between group comparisons. The measurement data with non-normal distribution are presented as median (25%, 75%) and a non-parametric independent sample *U*-test was performed for between group comparisons. Enumerative data were compared between the groups using the Pearson χ^2^-test, continuity corrected χ^2^-test, or Fisher's exact test. First, univariate analysis was performed to analyze the eye examination results and questionnaire survey results, independent variables with a *P* < 0.05 in univariate analysis were used to perform binary logistic regression analysis. The incidence of dry eye was considered the dependent variable. A *P* < 0.05 was considered statistically significant.

## Results

A total of 123 children and adolescent patients with DM are enrolled in the SCADE study. Because of the COVID-19 pandemic, only 40 of them (32.5%) were enrolled in the present study. The eye examination results of them are presented in Table 1. Among them, 55% (22/40) were girls and 45% (18/40) were boys. In 2018, none of the patients were diagnosed with dry eyer, and nine of them were diagnosed with dry eye by 2021, with both eyes meeting the criteria for dry eye. Among them, 77.8% (7/9) were girls, and 22.2% (2/9) were boys. The 3-year of rate of dry eye in children with DM was then calculated as 22.5% (9/40), with an average annual incidence rate of 7.5%.

**Table 1:** Characteristics and eye examination results of 40 children with DM.

**Characteristics**	**Subjects with dry eye**	**Subjects without dry eye**	**Statistics^#^**	***P***
Total number	9	31		
Gender/male (cases)	2	16	*x^2^* = 1.39	0.24
Age (years)	12.60 ± 2.69	12.56 ± 3.28	*t* = 0.03	0.97
The course of DM (years)	8.04 (6.25, 9.88)	4.76 (3.81, 7.28)	*U* = 219.5	0.01
BMI^*^ (kg/m^2^)	19.26 ± 2.32	20.23 ± 2.81	*t* = 0.94	0.35
OSDI (scores)	17.00 (16.50, 19.50)	10.00 (8.00, 12.00)	*U* = 278	< 0.01
HbA1c (mg/ml)	7.33 ± 1.26	8.22 ± 1.65	*t* = 1.00	0.33
Better-seeing eye BUT (s)	5.00 (4.50, 7.50)	10.00 (9.00, 12.00)	*U* = 15	< 0.01
Better-seeing eye Schirmer *I*-test (mm/5 min)	8.00 (4.50, 15.00)	18.00 (15.00, 25.00)	*U* = 42	< 0.01
Corneal hypoparesis (cases)	8	6	*x^2^* = 11.93	< 0.01
Corneal fluorescein staining/positive (cases)	5	1	*x^2^* = 11.16	< 0.01

* *BMI, Body mass index*.

# *t is an independent sample t-test, U is a non-parametric independent sample U-test, and x^2^ is a chi-square test*.

The results of univariate analysis for the incidence of dry eye in children with DM are presented in Tables 2, 3. Statistically significant differences were observed in the course of diabetes mellitus, corneal sensation (sensitive/less sensitive), eye pain (with/without), and dry eye in parents (with/without) in children with DM (*P* < 0.05). Further logistic regression analysis confirmed that the risk factor of the incidence of dry eye in children with DM was reduced corneal sensation (*P* < 0.05), as shown in Table 4.

**Table 2:** The questionnaire information of 40 children with DM.

**Items**	**Subjects with dry eye**	**Subjects without dry eye**	Statistics (*X*^**2**^)^*^	***P***
History of eye surgery/yes (cases)	0	0	—	—
History of eye trauma/yes (cases)	0	0	—	—
History of ocular medication/yes (cases)	2	3	1	0.32
Eating habits/meat (cases)	8	15	3.17	0.08
Allergic conjunctivitis/yes (cases)	2	3	0.13	0.72
Trichiasis/yes (cases)	0	0	—	—
Eye pain/yes (cases)	6	5	6.58	0.01^*^
Foreign body sensation in the eyes/yes (cases)	4	3	3.68	0.55
Vision fluctuation/yes (cases)	3	12	0.07	0.77
Refractive errors/yes (cases)	5	20	0.24	0.63
Use of contact lenses/yes (cases)	0	1	—	1
Reading and writing posture/right (cases)	5	10	1.62	0.2
Sleep disorders/yes (cases)	1	5	0.12	0.71
Using air conditioning/yes (cases)	8	24	0.57	0.45
Passive smoking/yes (cases)	0	2	—	1
Use electronic screen/mild (cases)	7	19	0.13	0.72
Eye strain/yes (cases)	7	20	0.56	0.46
Mental pressure/yes (cases)	3	13	0.22	0.64
Like blinking eyes/yes (cases)	3	7	0.43	0.51
Dry eye in parents/yes (cases)	7	10	4.2	0.04^*^

* *x^2^ is a chi-square test*.

**Table 3:** Better-seeing Eye examination results of 40 children with DM.

**Items**	**Subjects with dry eye**	**Subjects without dry eye**	**Statistics**	***P***
The number of children with DM	9	31		
Equivalent spherical lens degree (D)^*^	−1.40 ± 1.96	−2.72 ± 2.75	*t* = 1.33	0.19
Corneal endothelial cell count (/mm^2^)	3188.00 ± 426.69	3058.80 ± 378.66	*t* = 0.82	0.43
Ocular axial length (mm)	23.90 ± 0.81	24.57 ± 1.34	*t* = −1.42	0.16
Horizontal corneal radius of curvature, R1 (mm)	7.93 ± 0.27	7.95 ± 0.28	*t* = −0.2	0.85
Vertical corneal radius of curvature, R2 (mm)	7.64 ± 0.25	7.70 ± 0.30	*t* = −0.59	0.56
Corneal horizontal diameter (mm)	12.24 ± 0.41	12.13 ± 0.34	*t* = 0.76	0.46
Central corneal thickness (nm)	539.22 ± 33.16	552.55 ± 29.69	*t* = −1.16	0.25
Anterior chamber depth (mm)	3.65 ± 0.27	3.65 ± 0.26	*t* = 0	1
Lens thickness (mm)	3.52 ± 0.15	3.46 ± 0.24	*t* = 0.76	0.45

* *Equivalent spherical refraction = sphere + 1/2 cylinder*.

**Table 4:** Results of multivariate logistic regression analysis for dry eye in children with DM.

	**β**	***S.E*.**	***Wald***	***P***	***OR***	***95% CI***
Corneal sensation (decrease)	3.24	1.52	4.56	0.03	25.6	1.31–501.69
The course of DM (years)	0.59	0.32	3.3	0.07	1.8	0.96–3.38
Eye pain (yes)	2.51	1.5	2.8	0.09	12.27	0.65–231.48
Dry eye in parents (yes)	2.77	1.56	3.17	0.08	15.99	0.76–337.75
Constant	−9.71	3.49	7.74	0.01	0	

## Discussion

An increase in the number of children and adolescents with DM poses a serious economic burden on society; hence, measures to control the risk factors and reduce the morbidity rate of dry eye in them should be taken. According to previous study (13), when blood glucose keeps stable, the ocular surface impairment of patients may still aggravate in children with type 1 DM. The strict study design and uniform definitions and standards in our 3-year prospective study reveal the incidence and risk factors of dry eye in pediatric diabetes.

Previous studies have reported that the factors related to the prevalence of dry eye in adult patients with DM were age, sex, course of disease, corneal perception, fasting blood glucose (FBG), and glycosylated hemoglobin levels (HBA1c) (10, 15, 16). It is believed that the prevalence of dry eye in patients with DM increases with the increase of age, and the prevalence of dry eye in adult patients with DM is as high as 54.3% (8). Female patients with DM are more likely to suffer from dry eye (15). The prevalence of dry eye was higher in patients with high FBG and HBA1c (16).

The risk factors of high incidence of dry eye in children with DM is not the same as previous results in adults with DM. Previous cross-sectional study (10) showed that the corneal sensation was lower in children diagnosed with dry eye than those without. In the present prospective study, reduced corneal sensation is confirmed as the only statistically significant risk factor of dry eye. A clinical study conducted by Alise et al. showed that the degree of reduced corneal sensitivity in adult patients with DM was positively correlated with the severity of dry eye (17). The possible mechanisms involved may be that high-level glycosylated end metabolites may cause metabolic damage to the peripheral corneal nerve (18) and thus, reduce the corneal perceptual sensitivity and tear secretion in patients with DM (19–21). Additionally, corneal perception is positively correlated with blink reflex (22, 23) and the decrease in blink reflex can accelerate tear evaporation and lead to an increase in tear osmotic pressure.

Other factors may affect the incidence of dry eye (P close to 0.05) are the following: firstly, the course of DM: previous studies in adult populations have found that the longer the course of DM, the more severe damage of accessory lacrimal gland cells and the less the basic secretion of tears, and therefore, the increased prevalence of dry eye (24–26). Akinci et al. (9) believed that the duration of disease in patients with type 1 DM was the only factor related to tear secretion and tear film stability. Secondly, eye pain: Xiang et al. (27) believed that corneal neuralgia was a pre-clinical manifestation of dry eye. We believe that the cornea tissue damage may release inflammatory factors, stimulate corneal pain receptors, cause eye pain, and aggravate dry eye. Thirdly, family history: after further inquiry, we confirmed that none of the parents of the children with dry eye were DM patients or with other systemic diseases related to dry eye. Therefore, we speculated that this was related to the living environment, living habits, dietary structure, and micro-ecological structure of the children; however, further study is required to confirm these speculations.

Since all the subjects in this study were children aged 6–17 years, the age distribution difference and sample size were both small, and therefore, the age factor had no significant influence on the incidence of dry eye in children and adolescents with DM. In this study, all young patients wore insulin pumps, and then fasting and postprandial blood glucose were well-controlled. These may be the reason for no correlation between blood glucose and HbA1c levels and the incidence of dry eye in children with DM.

The shortcomings of this study are the following: no gold standard for the diagnosis of dry eye in children is currently available and therefore, we used the standard diagnosis criteria of dry eye in the adult population with diabetes mellitus. The participants of the SCADE cohort were students who had a tight course schedule, and furthermore, affected by the COVID-19 pandemic, many parents of the children included in the 2018 SCADE study withdrew from this investigation, resulting in a small sample size of the present study.

In conclusion, the incidence of dry eye in children with DM is high. Children with reduced corneal sensation, long course of diabetes, dry eye in parents, or eye pain should be examined for dry eye for early diagnosis and treatment to improve prognosis and the quality of life. Society, especially parents of these children, should be made aware of this through publicity and education. Simultaneously, it is necessary to establish a screening mode and better file management system, formulate prevention and treatment norms, and strengthen the scientific management of dry eye in children with DM.

## Data Availability Statement

The raw data supporting the conclusions of this article will be made available by the authors, without undue reservation.

## Ethics Statement

The studies involving human participants were reviewed and approved by the Institutional Review Committee and the Ethics Committee of the First People's Hospital Affiliated to Shanghai Jiao Tong University (approval number: 2018KY209). Written informed consent to participate in this study was provided by the participants' legal guardian/next of kin.

## Author Contributions

ZC: substantial contributions to the conception or design of the work or the acquisition, analysis, or interpretation of data for the work. HZ: drafting the work or revising it critically for important intellectual content, final approval of the version to be published, and agreement to be accountable for all aspects of the work in ensuring that questions related to the accuracy or integrity of any part of the work are appropriately investigated and resolved. YX, YQ, QL, ZX, LC, JS, SL, XQ, and CY: participate in survey and data collection. All authors contributed to the article and approved the submitted version.

## Funding

This work was supported by the Science and Technology Commission of Shanghai Municipality (Project No. 20DZ1100200), Shanghai Municipal Commission of Health (Public Health System Three-Year Plan-Key Subjects) (Project No. GWV10.1-XK7), The Project of Shanghai Shen Kang Hospital Development Center (Grant Nos. SHDC2020CR30538, SHDC2018110), Chinese National Nature Science Foundation (Project No. 82071012), Shanghai Engineering Research Center of Precise Diagnosis and Treatment of Eye Diseases, Shanghai, China (Project No. 19DZ2250100), and Shanghai General Hospital, Clinical Research (Project No. CTCCR-2018Z01).

## Conflict of Interest

The authors declare that the research was conducted in the absence of any commercial or financial relationships that could be construed as a potential conflict of interest.

## Publisher's Note

All claims expressed in this article are solely those of the authors and do not necessarily represent those of their affiliated organizations, or those of the publisher, the editors and the reviewers. Any product that may be evaluated in this article, or claim that may be made by its manufacturer, is not guaranteed or endorsed by the publisher.
